# A Novel Computational Model Predicts Key Regulators of Chemokine Gradient Formation in Lymph Nodes and Site-Specific Roles for CCL19 and ACKR4

**DOI:** 10.4049/jimmunol.1700377

**Published:** 2017-08-14

**Authors:** Mohammad Jafarnejad, David C. Zawieja, Bindi S. Brook, Robert J. B. Nibbs, James E. Moore

**Affiliations:** * Department of Bioengineering, Imperial College London, London SW7 2AZ, United Kingdom;; † Department of Medical Physiology, Texas A&M Health Science Center, Temple, TX 76504;; ‡ School of Mathematical Sciences, University of Nottingham, Nottingham NG7 2RD, United Kingdom; and; § Institute of Infection, Immunity and Inflammation, College of Medical, Veterinary and Life Sciences, University of Glasgow, Glasgow G12 8TA, United Kingdom

## Abstract

The chemokine receptor CCR7 drives leukocyte migration into and within lymph nodes (LNs). It is activated by chemokines CCL19 and CCL21, which are scavenged by the atypical chemokine receptor ACKR4. CCR7-dependent navigation is determined by the distribution of extracellular CCL19 and CCL21, which form concentration gradients at specific microanatomical locations. The mechanisms underpinning the establishment and regulation of these gradients are poorly understood. In this article, we have incorporated multiple biochemical processes describing the CCL19–CCL21–CCR7–ACKR4 network into our model of LN fluid flow to establish a computational model to investigate intranodal chemokine gradients. Importantly, the model recapitulates CCL21 gradients observed experimentally in B cell follicles and interfollicular regions, building confidence in its ability to accurately predict intranodal chemokine distribution. Parameter variation analysis indicates that the directionality of these gradients is robust, but their magnitude is sensitive to these key parameters: chemokine production, diffusivity, matrix binding site availability, and CCR7 abundance. The model indicates that lymph flow shapes intranodal CCL21 gradients, and that CCL19 is functionally important at the boundary between B cell follicles and the T cell area. It also predicts that ACKR4 in LNs prevents CCL19/CCL21 accumulation in efferent lymph, but does not control intranodal gradients. Instead, it attributes the disrupted interfollicular CCL21 gradients observed in *Ackr4*-deficient LNs to ACKR4 loss upstream. Our novel approach has therefore generated new testable hypotheses and alternative interpretations of experimental data. Moreover, it acts as a framework to investigate gradients at other locations, including those that cannot be visualized experimentally or involve other chemokines.

## Introduction

Immunosurveillance and immune responses depend on precisely coordinated leukocyte migration. This is largely orchestrated by chemokines, which are sensed by heptahelical G protein–coupled chemokine receptors on leukocytes (1, 2). Chemokines induce various types of cell migration, particularly haptotaxis and chemotaxis, which involve directed cell movement up a concentration gradient. The mechanisms that control the formation and regulation of chemokine gradients in vivo are poorly understood but likely involve many interrelated biochemical and physical processes (3–5). Understanding how these processes are integrated and modulated remains a major challenge in chemokine biology. In this article, we develop a computational model of chemokine transport within the lymph node (LN) to explore the contribution of distinct physical and biological phenomena to chemokine gradient formation.

**FIGURE 1.**
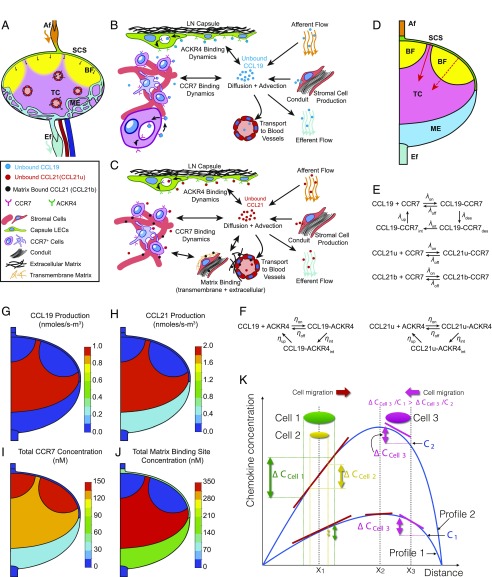
Methodological information on building the model. (**A**–**C**) Schematic of LN with CCL19 and CCL21 interactions. (A) Schematic showing lymph entry into SCS from the Af, after which it can percolate into BFs and T cell paracortex (TC) and be exchanged with blood vessels; or flow via the medulla (ME) into the Ef. (B and C) Interactions of CCL19 (B) or CCL21 (C): each arrow indicates a reaction or transport term in the model. (**D**) Idealized LN geometry used in the computational model with regions color coded. Abbreviations as in (A). Red arrows indicate the locations where concentration gradients were measured. (**E** and **F**) Reaction diagrams for chemokine interactions with CCR7 and ACKR4. (E) CCR7 is desensitized and internalized after binding CCL19, after which CCR7 returns to the cell surface and CCL19 is degraded. CCL21–CCR7 interactions use ligand–receptor models without desensitization or internalization. (F) Binding of CCL19 or CCL21u to ACKR4 results in chemokine internalization and degradation, and subsequent recycling of ACKR4 to the cell surface. (**G**–**J**) Distribution of CCL19/CCL21 production, CCR7 density, and matrix binding site density in the PLN. Assuming homogenous cell distribution within each PLN region, CCL19 production (G), CCL21 production (H), CCR7 concentration (I), and matrix binding site concentration (J) were calculated for each region using Eq. 16 in Supplemental Fig. 1. Production rates or concentrations are color coded according to scales to the right of each image. (**K**) Sample concentration profiles illustrating how gradients and concentration differences across cells were calculated. Chemokine concentration profiles (blue curves) are plotted as a function of distance along a predefined direction [e.g., red arrows in (D)]. The gradient is the slope of the profile at a particular location (red and magenta lines). If the concentration profile is nonlinear, then the magnitude and/or sign of the gradient vary with position. A cell’s size determines the concentration differences it can sense, e.g., cell 1 at position x_1_ senses a larger concentration gradient (ΔC_Cell 1_) than a smaller cell, cell 2, at the same location (ΔC_Cell 2_). In different profiles there may be the same concentration difference across a cell (e.g., the magenta gradients in profiles 1 and 2 are of the same magnitude, so ΔC_Cell 3_ would have the same value in both cases). However, if these are normalized with respect to the absolute concentration values at one edge of the cell (e.g., C_1_ and C_2_), then the percentage concentration difference will be greater for the cell in profile 1 than in profile 2. We therefore also plot the percentage concentration difference across a cell. Directionality (i.e., the direction in which a cell migrates) depends on the sign of the gradient. Given that leukocytes typically migrate up concentration gradients, cells will be predicted to migrate in the positive direction (e.g., from x_1_ to x_2_) where gradients are positive (red lines), and in the opposite direction (e.g., from x_3_ to x_2_) where they are negative (magenta lines).

Evidence of extracellular chemokine gradients in vivo is mostly indirect and based on visualizing leukocyte behavior by intravital microscopy. However, because of its abundance and high affinity for extracellular matrix (ECM), gradients of CCL21 have been directly detected in tissues, including in interfollicular regions (IFRs) and B cell follicles (BFs) of LNs (Fig. 1), where CCL21 concentration increases toward the T cell area (TC) in the paracortex (6–8). CCR7, the receptor for CCL21, is critically involved in cell migration in LNs controlling lymphocyte entry from the blood, intranodal T cell motility, T cell retention, and the migration of B cells in the BF to the BF–TC boundary (6, 9, 10). It is also required for dendritic cells (DCs) to move from the subcapsular sinus (SCS) into the IFR, and for their subsequent directional migration to the TC (11). CCR7 also binds CCL19, which is produced primarily by stromal cells in the TC. CCL19 lacks the highly charged extended C terminus present in CCL21, resulting in a much lower affinity for ECM (12–15). Consequently, CCL19 and CCL21 will differ markedly in their distribution and retention in LNs. CCL21 is the dominant CCR7 ligand, and although CCL19 can direct chemotaxis through CCR7 in vitro, it is dispensable for lymphocyte and DC entry into LNs (16–18). However, it can contribute to T cell homeostasis (18) and is thought to control leukocyte behavior inside LNs (19). Interestingly, CCL19 and CCL21 elicit different intracellular signals through CCR7, and only CCL19 drives CCR7 desensitization and internalization of ligand–receptor complexes (19–24).

CCL19 and CCL21 also bind to the atypical chemokine receptor ACKR4 (also known as CCRL1 or CCX-CKR) (25). It is expressed by lymphatic endothelial cells (LECs) on the SCS ceiling (7) and by stromal cells in nonlymphoid tissues, such as keratinocytes and some LECs in skin (17, 26). Although structurally related to CCR7, ACKR4 cannot drive cell migration but can mediate chemokine internalization and destruction (7, 26, 27). This scavenging activity regulates CCR7-directed DC migration in vivo (7, 17, 26). In resting *Ackr4*-deficient mice, CCL21 gradients in the IFR of skin-draining LN (SLN) are swamped by excess CCL21 and DC egress from the SCS is delayed (7). During cutaneous inflammation, scavenging by ACKR4 prevents CCL19 from interfering with the CCR7-mediated detection of CCL21 which is required to direct DC trafficking out of the skin to the SLNs (17).

CCR7-dependent migration in vivo is ultimately dependent on the precise distribution of extracellular CCL19 and CCL21. This will be influenced by many biochemical and physical processes including the rate or site of production; diffusion in tissue; interaction with ECM; internalization by CCR7; scavenging by ACKR4; and fluid flow in blood vessels, lymphatic vessels, and the interstitial space. To explore this complex process, we have built all these factors into a novel three-dimensional computational model of chemokine distribution in skin-draining popliteal LNs (PLNs) by integrating multiple biochemical processes into our established model of PLN lymph flow and fluid exchange (28). This has allowed us to predict how distinct physical and biochemical processes shape chemokine gradients, and determine which of these processes are likely to be of particular biological importance. It has also guided the formulation of novel testable hypotheses to focus future experimentation. Moreover, this comprehensive modeling approach can be used as a framework to investigate gradients at other locations and involving other chemokines.

## Materials and Methods

We have developed a mathematical model that accounts for the key biological and physical processes that determine chemokine gradients in LNs, and solved the resulting complex system of equations computationally. To do this, we have coupled equations describing chemokine reactions to our previous model of fluid flow in the LN (28). Application of mathematical models to biology using this general approach has a long history (29–31), and such models have been developed in previous studies to describe general chemokine behavior (4, 5, 29–31). The key interactions regulating the distribution of extracellular CCL19 and CCL21, and which are modeled in this study, are summarized in Fig. 1A–C.

### Modeling LN fluid transport

The model of lymph flow through the PLN is based on our published work (28). Briefly, we constructed an idealized three-dimensional geometry based on the general features of a mouse PLN (∼1 mm in diameter), including an afferent vessel (Af), an efferent vessel (Ef), and the different zones of the node (SCS, BFs, TC, medulla) (Fig. 1D). Computer-aided modeling software (ANSYS, Canonsburg, PA) was used to generate a computational mesh and solve the equations of fluid flow. Wall shear stress along the SCS was used as the criterion for mesh refinement (28). The Navier–Stokes equations were used to describe flow in the fluidic regions (i.e., Af, SCS, and Ef), and the Darcy law with the Brinkman term was used in the porous regions (i.e., BF, TC, and medulla). The fluid exchange between blood vessels and lymphatic compartments was modeled using the Starling equation. All fluid flow parameters were the same as those in our previous work (28).

### Chemokine transport

The general description of chemokine dynamics consists of two partial differential equations and six ordinary differential equations (ODEs). The concentration evolution of transportable species (unbound CCL21 [CCL21u] and CCL19) are governed by partial differential equations representing diffusion, advection, and reactions (binding, uptake, and scavenging), as shown in Supplemental Fig. 1, Eqs. 1 and 2. Concentrations of receptor- and ECM-bound chemokines, as well as receptor densities, are governed by ODEs (Supplemental Fig. 1, Eqs. 3–8). These quantities vary among LN regions but are not transported by diffusion or advection.

### CCL21 binding to matrix

The process of CCL21 binding to, and unbinding from, ECM is modeled with first-order reaction kinetics (Supplemental Fig. 1, Eqs. 1, 3), where the binding rate constants *k*_1_ and *k*_2_ were measured by Shields et al. (31) for CCL21 binding to perlecan as 0.000093 nM^−1^s^−1^ and 0.00012 s^−1^, respectively. The maximum number of CCL21 binding sites associated with each cell was assumed to be 10^6^, which is similar to Matrigel containing 1% perlecan (Supplemental Fig. 2) (31). The spatially averaged ECM binding site density in each region of the LN was calculated according to the process described below in *Parameter estimation*.

### CCR7 binding dynamics

CCR7 can bind to CCL19 or CCL21, but only CCL19 efficiently induces desensitization and internalization of CCR7 (19–24) (Fig. 1E). For CCL19/CCR7 binding dynamics, we have employed a simplified version of a previous model (29) as given in Eqs. 4–6 in Supplemental Fig. 1. We have assumed CCL21u and ECM-bound CCL21 (CCL21b) bind to, but do not desensitize or internalize, CCR7 (Fig. 1E). They are thus modeled by Eqs. 7 and 8 in Supplemental Fig. 1. The total number of CCR7 molecules per cell (in all their different states) is assumed to be constant (Supplemental Fig. 1, Eq. 9). The total number of receptors in each computational element was calculated based on the continuum assumption explained in *Parameter estimation*, and CCR7 expression values are obtained from the Immunological Genome Project (ImmGen) database (Supplemental Fig. 3A) (32, 33). CCR7 is only located on cell membranes, but we have assumed that CCR7 in each computational element is uniformly distributed. On (λ_on_ = 0.001 nM^−1^s^−1^) and off (λ_off_ = 0.005 s^−1^) binding rates to CCR7 are similar for CCL21 and CCL19 and are calculated from dissociation constants determined experimentally (27, 29, 30, 34, 35). Rate constants for desensitization (λ_des_ = 0.003 s^−1^), internalization (λ_int_ = 0.0005 s^−1^), and recycling (λ_up_ = 0.000375 s^−1^) of CCL19–CCR7 were calculated from curve fits of experimental data (29, 30) (Supplemental Fig. 2). The maximum number of CCR7 molecules per cell is estimated to be 30,000 in the baseline case.

### ACKR4-mediated chemokine scavenging

In LNs, only LECs on the SCS ceiling express ACKR4 (7). A receptor internalization model was therefore adopted (Fig. 1F) to describe ACKR4 dynamics and CCL19/CCL21u scavenging on the capsule boundary as given by four additional ODEs (Supplemental Fig. 1, Eqs. 11–14). The total number of ACKR4 molecules in all its different states per LEC is assumed to be constant (Supplemental Fig. 1, Eq. 15) and 30,000 in the baseline case. From the number of LECs we estimated to be in the SCS, half were assumed to be on the ceiling, which leads to an average cell diameter of ∼25 μm. The same LEC density was assumed for the capsule on the medulla. All other molecules are assumed to have zero flux at the surface of the capsule. Based on our previous studies of ACKR4-mediated CCL19 scavenging (27), we assumed that receptor resurfacing is the limiting factor and then set the rest of the reaction parameters of the model to have similar scavenging rates (η_in_ = 1.0 s^−1^ and η_up_ = 0.002 s^−1^). Additionally, we measured the dissociation constant to be 4.5 nM (27) and set the on and off rates accordingly (η_on_ = 0.5 nM^−1^s^−1^ and η_off_ = 2.25 s^−1^).

### Chemokine transport to blood vessels

Very low concentrations of CCL19 and CCL21 are present in plasma at physiological conditions (36, 37). In mice, chemokines injected in the foot pad appear on the luminal surface of PLN blood vessels within 90 min (38). Chemokine transport to blood vessels can occur directly within LNs by convection from fluid exchange and by diffusion due to concentration difference. These are indicated by the first and second terms, respectively, in Eq. 10 in Supplemental Fig. 1. Under basal conditions, some lymph fluid is transported from lymphatic compartments to blood vessels in the LN, for which the fluid exchange parameters from the Starling equation are assumed to be the same as in our previous work (28, 39, 40). CCL19 and CCL21 (8.8–12.2 kDa) molecules are estimated to have approximately half the radius of albumin (68 kDa) (41), giving an estimated chemokine permeability of 5 × 10^−7^ cm/s, an order of magnitude greater than albumin (42).

### Boundary conditions

Eqs. 1–9 in Supplemental Fig. 1 were solved in the steady-state form (without the transient terms on the left-hand side). To initiate the numerical solution, starting values of zero were assumed for all variables. Chemokine concentration was assumed to be constant and equal to zero at the Af inlet and the Ef outlet, unless otherwise indicated. A quarter of the LN was modeled with symmetry boundary conditions on the two sidewalls. Steady-state versions of the ACKR4 Eqs. 11–15 in Supplemental Fig. 1 were solved at the capsule boundary to determine the scavenging flux for CCL19 and CCL21u, and were applied as boundary conditions on the capsule ceiling (Supplemental Fig. 1, Eq. 16). The computational code was then run until the solution converged to a steady state.

### Parameter estimation

Because of the complexity of having different cell types in multiple regions in the LN, we have used gene expression data from ImmGen (Supplemental Fig. 3A) to identify the dominant expression pattern. This leads to a set of simplifying assumptions that greatly reduce the number of free parameters in the model. We have assumed that the cell types are homogenously distributed within each of the regions (Supplemental Fig. 3B) and that the protein levels are proportional to the expression values obtained from ImmGen (Supplemental Fig. 3A) (32, 33). With this we can calculate the parameters of interest by multiplying the total cell number types by the percentage of cells in the particular region and the expression level, normalized by the computational element volume size (Supplemental Fig. 1, Eq. 17) (43–45). For example, for the total number of CCR7 molecules in the TC, we multiply the total number of T cells, *N_k_* (∼10^6^ in PLN; Supplemental Fig. 3B), by the percentage of T cells (cell type *k*) in the TC (region *j*), *P_k,j_* (0.9, Supplemental Fig. 3B); then multiply this by the normalized CCR7 expression value for T cells, *EV_X,k_* (0.5, Supplemental Fig. 3A), and maximum possible CCR7 per cell, *X*^max^ (30,000). We then divide by the Avogadro number, *N_A_*, to find the total moles of CCR7 on T cells in the TC. Assuming homogenous distribution, we scale the total moles with the volume of the computational element (*Vol_i_*) with respect to the volume of the TC (*Vol_j_*) to find the moles of CCR7 in that element. Summing all the moles of CCR7 from different cell types, *k*, (B cell, T cell, DC, macrophage, LEC, blood endothelial cell, and fibroblastic reticular cell) provides an estimate of total moles of CCR7 in that computational element. Similarly, we estimated the rate of CCL19 production (Fig. 1G), the rate of CCL21 production (Fig. 1H), CCR7 density (Fig. 1I), and ECM binding site density (Fig. 1J).

### Parameter variation analysis

Latin hypercube sampling was used to test the robustness of the model and to investigate the effect of variation in seven biological parameters on gradients of CCL21 and CCL19. These were selected because of their potential biological importance, previous investigations in animal models, and the level of confidence in their estimation from previously published data. The input parameters and bounds on their distribution are shown in Supplemental Fig. 3C. From simulations with 100 different input parameter combinations, we quantified the slopes of the CCL19/CCL21b concentration profiles (along the arrows shown in Fig. 1D), the total amounts of CCL19 and CCL21 in the whole LN, and Ef concentrations of CCL19 and CCL21. The volume integral of the concentration over LN volume was used to calculate total CCL19 and CCL21 (CCL21u and CCL21b). Partial rank correlation coefficients (PRCCs) were calculated to identify positive and negative correlations between the main input parameters and the outputs (46, 47). The Student *t* test was used to determine the significance of the PRCCs. MATLAB R2013a (MathWorks) was used for PRCC calculation and statistical analysis.

### Calculation of concentration difference across cells

Spatial concentration differences were calculated based on concentrations *C_i_* (*i* = 1..N) at N locations along the direction of interest. The difference across a cell is *C_i+1_ − C_i_* for *i* = 1..N−1. As previously (8), each location was separated by 9, 18, or 36 μm to cover a range of cell sizes from small lymphocytes to larger DCs (Fig. 1K). Along the arrows (Fig. 1D), the baseline gradients were resampled at a spatial resolution of 4.5 μm and low-pass filtered.

## Results

### Baseline computational simulations generate intranodal chemokine gradients

**FIGURE 2.**
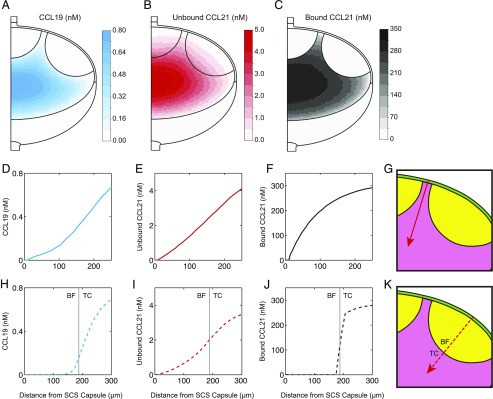
CCL19 and CCL21 form gradients in the IFR and BF–TC border of the LN. Contours of concentrations of CCL19 (**A**), CCL21u (**B**), and CCL21b (**C**) in an LN with an idealized geometry. The depth of color in (A)–(C) gives an indication of concentration, according to the scales shown in each panel. The IFR (**D**–**F**) and BF–TC border (**H**–**J**) concentration profiles are quantified along the red arrows indicated (**G** and **K**) for each of the corresponding contours. All profiles start from the ceiling of the SCS and include the sinus (10 μm height). Vertical solid lines in (H)–(J) show the location of BF–TC border at 188 μm from the SCS.

In baseline computational simulations of intranodal steady-state chemokine distribution, the highest concentrations of CCL19, CCL21b, and CCL21u occurred in the center of the TC (Fig. 2A–C). CCL21b, at a maximum concentration of 306 nM, was much more abundant than CCL19 or CCL21u (maximum concentrations of 0.9 and 5.2 nM, respectively). Consistent with experimental observations (7), gradients of CCL21 (CCL21u and CCL21b) were observed in the IFR. The model also predicts that a CCL19 gradient forms here. The CCL19 and CCL21u gradients had a nearly uniform magnitude (∼0.003 and ∼0.018 nM/μm, respectively), whereas the CCL21b gradient was highest at the border with the SCS (3.7 nM/μm) and dropped by nearly an order of magnitude, 200 μm from the SCS floor (Fig. 2F). Nonetheless, the IFR gradient of CCL21b was at least 17 times larger than those of CCL19 or CCL21u.

Chemokine gradients were also present across the BF–TC boundary and into the BF (Fig. 2H–K). CCL21b and CCL19 were virtually absent from BFs so large gradients formed at the BF–TC boundary. The CCL19 gradient was at most 0.004 nM/μm, whereas that of CCL21b was 10 nM/μm over a 25 μm region. The simulations also predicted the existence of gradients of CCL21u (0.008–0.023 nM/μm) that emanated from the TC but extended deeper into the BF than the CCL19 and CCL21b gradients, virtually reaching the SCS. These were similar in shape to those present in the IFRs. A CCL21 gradient leading to the BF–TC border has been detected immunohistochemically in BFs (6).

Thus, baseline simulations predict gradients of CCL19, CCL21u, and CCL21b at two locations in LNs. The gradients of CCL21 resemble those that have been experimentally observed, building confidence in the ability of the computational model to accurately predict intranodal chemokine distribution.

### Concentration differences across representative cell sizes

**FIGURE 3.**
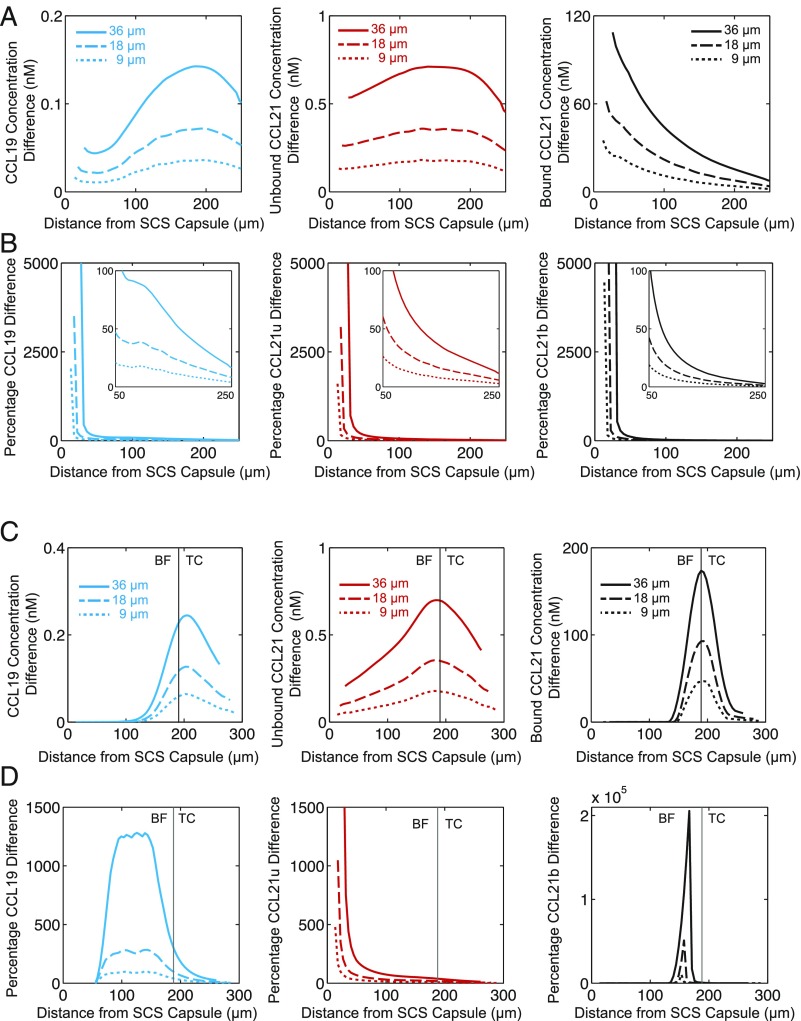
Chemokine concentration difference across cells in the IFR and at the BF–TC border. Concentration differences across a cell were calculated for CCL19 (blue), CCL21u (red), and CCL21b (black) using the baseline chemokine distribution data (Fig. 2) for cells with a diameter of 9, 18, and 36 μm with their leading edge pointing away from the SCS. (**A** and **B**) IFRs. (**C** and **D**) BF, BF–TC border, and TC. The data are presented as a total concentration difference (A and C) and as a percentage concentration difference (B and D). Thirty six–micrometer cell (solid lines), 18-μm cell (dashed lines), and 9-μm cell (dotted lines). Vertical solid lines in (C) and (D) show the location of BF–TC border at 188 μm from the SCS.

Next, we considered how these gradients might be interpreted by leukocytes. Directed migration depends on a cell sensing a chemokine concentration difference between its leading and trailing edges. This is determined computationally by the magnitude of the gradient and the cell size (Fig. 1K), and was calculated across cells with 9, 18, or 36 μm diameters with their leading edge pointing toward the TC (Fig. 3). We considered cells in IFRs (from the SCS ceiling to the TC) (Fig. 3A, 3B), and those in BFs, across the BF–TC border and into the TC (Fig. 3C, 3D). The data are presented both as a total concentration difference (Fig. 3A, 3C) and as a percentage concentration difference (Fig. 3B, 3D). As expected, concentration differences were always positive for all chemokines, and there was a linear relationship between cell size and concentration difference, with larger differences seen by larger cells. The absolute concentration differences of CCL19 and CCL21u did not vary much within different regions of the IFR for a cell of a given size (Fig. 3A). Those for CCL21b were of a much higher magnitude (up to ∼110 nM at the SCS floor), but they got substantially smaller with increasing distance from the SCS (Fig. 3A). The profiles of the percentage concentration differences were broadly similar for CCL19, CCL21u, and CCL21b, with by far the greatest difference seen for all at the SCS floor due to their very low concentrations in the SCS (Fig. 3B).

Large CCL21b concentration differences were present around the BF–TC border (Fig. 3C, 3D). However, concentration differences in CCL19, and particularly CCL21u, were present throughout the BF in addition to the BF–TC border (Fig. 3C, 3D). The largest percentage of CCL21u concentration differences were just underneath the floor of the SCS, whereas regions of the BF closer to the BF–TC border showed the largest percentage of CCL19 concentration differences (Fig. 3D). We also considered cells in the TC and at the BF–TC border with their leading edge pointing toward the SCS: as expected, concentration differences were negative for all chemokines and became increasingly negative as cells in the TC were positioned closer to the BF–TC border (data not shown).

Thus, IFR gradients appear capable of directing cells out of the SCS and then deeper into the TC. Cells in the BF have the potential to be drawn toward and across the BF–TC border in response to CCR7 ligands, but the precise location of the cell will likely determine whether it responds to CCL19, CCL21u, and/or CCL21b. Conversely, cells in the TC will encounter increasingly negative concentration differences as they approach the BF–TC border.

### Regional variations in chemokine–CCR7 complexes

**FIGURE 4.**
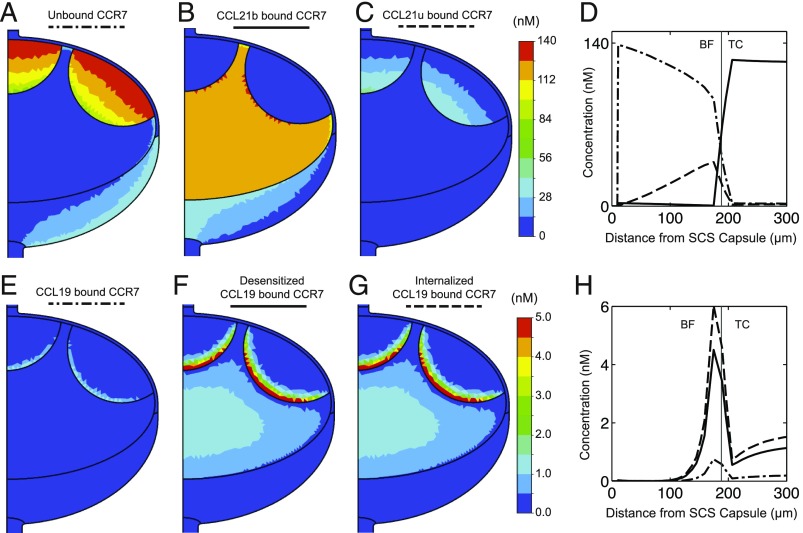
Regional variations in the occupancy, internalization, and desensitization of CCR7. Contours of concentrations (in nM) of unbound CCR7 (**A**); CCR7 bound to CCL21b (**B**), CCL21u (**C**), or CCL19 (**E**); or CCR7 desensitized (**F**) or internalized (**G**) as a consequence of binding to CCL19. The concentrations of each form of CCR7 at specific parts of the LN are shown in different colors according to the scales shown to the right of panels (A)–(C) or (E)–(G). During the simulations, it was assumed that CCR7 is absent from Af, SCS, and Ef, and that the total concentration of CCR7 is constant in each region. (**D** and **H**) Concentration profiles from the SCS through the BF and across the BF–TC border (i.e., along the dashed red arrow shown in Fig. 1D). The graphs in (D) and (H) have been generated from the contour plots in (A)–(C) and (E)–(G), respectively, and the identity of the lines in these graphs is indicated above the contour plots.

The analysis above gives a clearer picture of how CCR7^+^ cells might sense gradients predicted by the model, but very high or very low levels of receptor occupancy, along with receptor desensitization and internalization, have implications for the ability of gradients to induce migration. Therefore, we next examined CCR7 occupancy and CCL19-driven CCR7 desensitization/internalization in the LN under baseline conditions (Fig. 4). Based on the distribution of CCR7^+^ leukocytes in LNs, CCR7 was modeled as being absent from the SCS, low in the medulla, and abundant in the TC and BF (Fig. 1I).

Virtually all CCR7 molecules in the TC and IFR were predicted to be occupied by chemokine, the vast majority by CCL21b (Fig. 4B), with only ∼2% available for CCL21u or CCL19 (Fig. 4C, 4E); although there was a small amount of unbound CCR7 present at the border between the SCS floor and the IFR (Fig. 4A). Exposure to CCL19 in the core of the TC was predicted to result in the internalization and desensitization of virtually none (<1%) of the available CCR7 molecules (Fig. 4F, 4G).

In the BF, the absence of CCL21b meant that more CCR7 here was unoccupied and therefore available for binding to CCL21u or CCL19 originating from the TC (Fig. 4C–E). The number of receptors occupied by CCL21u and CCL19 increased with proximity to the BF–TC border, before plummeting abruptly once the border was crossed. The peak concentrations of CCL21u–CCR7 (Fig. 4C, 4D) and CCL19–CCR7 (Fig. 4E, 4H) complexes near the BF–TC border were 44.2 and 1.4 nM, respectively. Conversely, unbound CCR7 was at its highest at the edge of the BF adjacent to the SCS (Fig. 4A, 4D), although some CCL21u–CCR7 complexes were still present even in this region of the BF (Fig. 4C, 4D). Interestingly, there was a striking peak of CCL19-driven CCR7 desensitization and internalization in the BF adjacent to the BF–TC border; this was absent from regions deeper in the BF, and four- to fivefold higher than in the TC (Fig. 4F–H).

Thus, the model predicts that the intranodal distribution of CCR7 ligands leads to wide variation in CCR7 occupancy and desensitization/internalization between, and within, distinct microanatomical niches. This has implications for how these chemokine gradients might direct and control cell migration at specific locations (see *Discussion*).

### Parameter variation analysis

**FIGURE 5.**
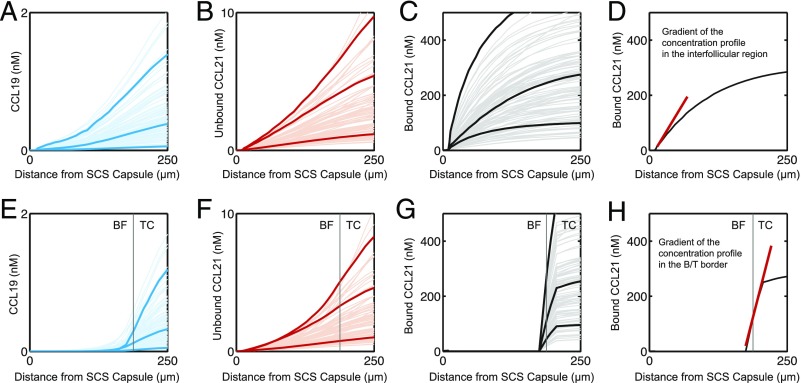
PVA. One hundred simulations were run containing randomly generated variations in key parameters controlling intranodal gradient formation. (**A**–**C**) Concentration profiles of CCL19 (A), CCL21u (B), and CCL21b (C) in the IFR. (**E**–**G**) Concentration profiles of CCL19 (E), CCL21u (F), and CCL21b (G) at the BF–TC border. The three bold lines in (A)–(C) and (E)–(G) are unrelated representative concentration profiles that have been highlighted to illustrate the types of gradients observed. (**D** and **H**) Individual representative CCL21b concentration profiles from the IFR (D) and BF–TC border (H) overlaid with red lines that show where the gradient magnitude was measured for the correlation analysis (Table I).

Parameter variation analysis (PVA) provides a means of evaluating variations and uncertainties in input parameters that may be key regulators of a model’s outputs and, by extension, the physiological process being modeled. With this approach, we determined which parameters are key in determining the intranodal gradients of CCL19, CCL21u, and CCL21b generated by our model. One hundred simulations were run in which random variations were simultaneously introduced into each of the following parameters: intranodal production of CCL19 or CCL21, the number of CCR7 or ACKR4 receptors (on cells known to express these receptors), the concentration of ECM binding sites, and the effective diffusion coefficient of each chemokine (*D_eff_*). The upper and lower limits used in these simulations are detailed in Supplemental Fig. 3C. We examined the shape and directionality of chemokine gradients formed in these simulations (Fig. 5A–C, E–G) and explored whether correlations existed between any of the randomly generated variations and either 1) the amount of chemokine in the LN or efferent lymph (CCL19 or CCL21u and CCL21b), or 2) the magnitude of the gradient across the BF–TC border (Fig. 5H) or in the IFR immediately underneath the SCS (Fig. 5D).

As expected, CCL21b was much more abundant than CCL19 and CCL21u in all simulations (Fig. 5). When there were elevated quantities of CCL21b in the IFR, a step gradient formed at the floor of the SCS (Fig. 5C). Importantly, the concentration profiles clearly showed that although gradient magnitude varied considerably across the simulations, the intranodal gradients of CCL19, CCL21u, and CCL21b maintained their directionality, increasing in concentration from the floor of the SCS into the TC, and from the BF across the BF–TC border (Fig. 5). However, various correlations were found between the randomly modified parameters and either the amount of chemokine (in the LN or efferent lymph) or the magnitude of the gradients immediately underneath the SCS or across the BF–TC border (Table I).

**Table I.** Results of PVA are summarized for key model outputs (for CCL19 and CCL21) and significantly correlated input parameters

Input Parameter	Output Parameter							
	Interfollicular CCL19 Gradient	Interfollicular CCL21b Gradient	BF–TC Border CCL19 Gradient	BF–TC Border CCL21b Gradient	Total CCL19 in LN	Total CCL21 in LN	Efferent CCL19 Concentration	Efferent CCL21 Concentration
Matrix binding sites		+ + +	+ + +	+ + +	+ + +	+ + +	+ + +	
CCL19 production	+ + +		+ + +		+ + +		+ + +	
CCL21 production		+ + +		+ + +	+ +	+ + +	+ + +	+ + +
CCR7 per cell	− −	+			− − −		− − −	+
ACKR4 per cell							− − −	− − −
Effective diffusivity	− − −	− − −	− − −	− −	− − −	− − −	+ + +	+ + +

Significant PRCC values are as follows: +/−, 0.001 < *p* < 0.01; + +/− −, 0.0001 < *p* < 0.001; + + +/− − −, *p* < 0.0001.

First, there was a strong correlation between the ECM binding site concentration and the magnitudes of the CCL21b gradients under the SCS and across the BF–TC border. In addition, lower matrix binding site concentrations correlated with decreasing amounts of CCL21 and CCL19 in the LN. This was positive for CCL21, because decreased ECM reduced CCL21b accumulation, but negative for CCL19 because a drop in CCL21b increased free CCR7 to enhance CCR7-mediated CCL19 internalization and degradation. For the same reason, the level of CCL21 production positively correlated with both total CCL21 and total CCL19 in the LN, whereas production of CCL19 only correlated with levels of CCL19. Similarly, increased ECM binding sites, CCL21 production, or CCL19 production correlated with the accumulation of CCL19 in efferent lymph. Again, because of CCR7-mediated CCL19 internalization, there were strong negative correlations between the quantity of CCR7 in the LN and CCL19-related parameters (i.e., gradient magnitude, total amount in LN and efferent lymph). Correlations between the CCR7 level and CCL21-related parameters were less apparent, although weak positive correlations existed between CCR7 and the IFR CCL21 gradient magnitude, and the amount of CCL21 in efferent lymph. There were strong negative correlations between the effective diffusivity of CCL19 and CCL21 and the magnitude of their respective gradients under the SCS and across the BF–TC border. At the same time, higher effective diffusivity reduced the total amount of chemokines in the LN, while increasing their presence in efferent lymph. Finally, the number of ACKR4 molecules per LEC on the ceiling of the SCS did not significantly correlate with changes in gradients of CCL19 and CCL21, or the total amount of chemokine in the LN, but did negatively correlate with the concentration of CCL19 and CCL21 in efferent lymph.

These data demonstrate that gradient directionality is a stable output from the model. However, variations in key biological or physical parameters affect the magnitudes of these gradients and/or the total amount of chemokine in the LN or efferent lymph.

### Role of ACKR4 in LN versus peripheral tissue

**FIGURE 6.**
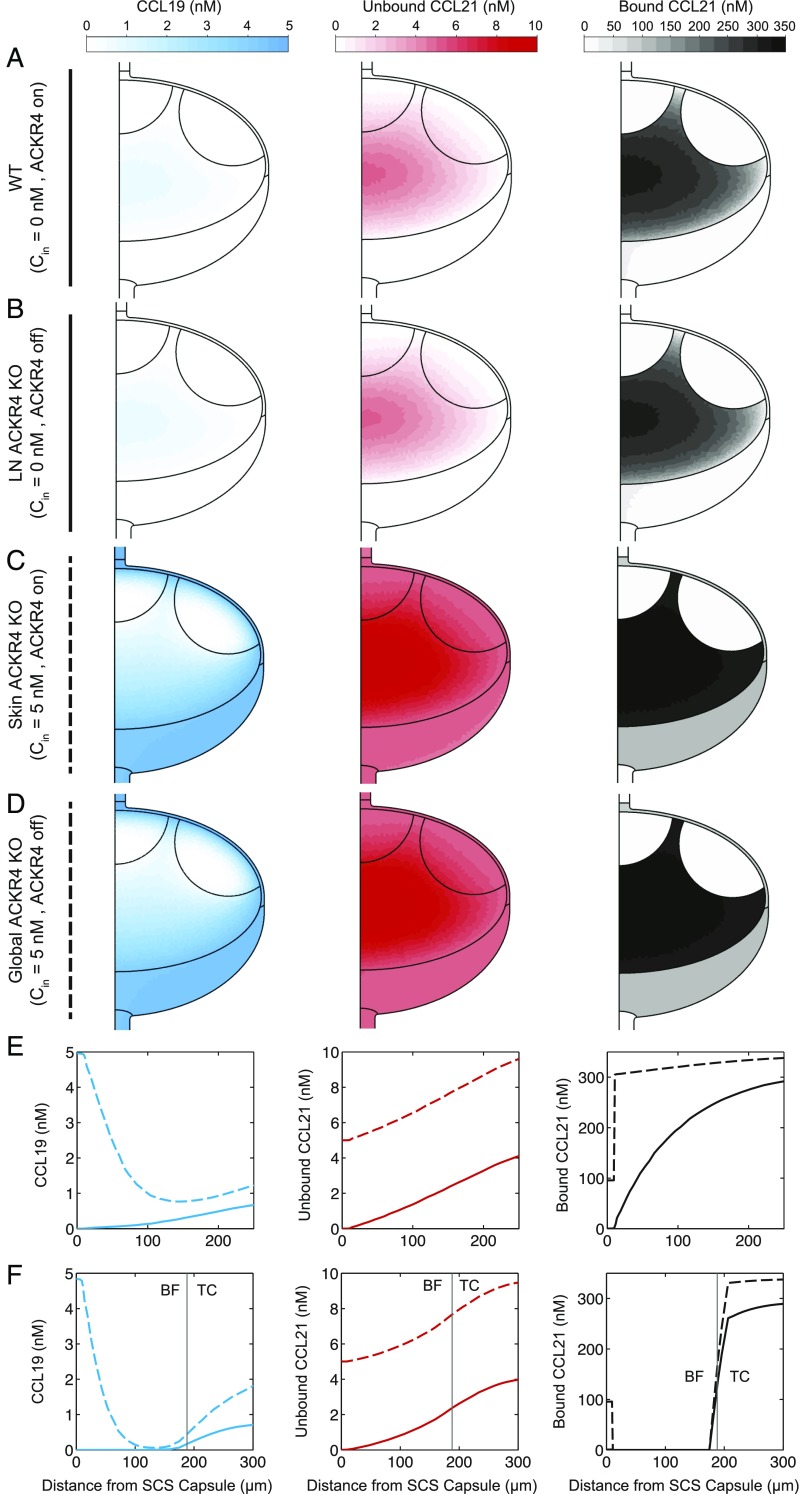
Simulations of the impact of *Ackr4* deficiency in SCS and/or skin on intranodal chemokine distribution. Contours (**A**–**D**) and plotted gradients (**E** and **F**) in SLN of CCL19 (left column, blue), CCL21u (middle column, red), and CCL21b (right column, black) in the following simulations: (A) WT control [solid lines in (E) and (F)], (B) *Ackr4* deficiency in the LN only (LN ACKR4 KO) [solid lines in (E) and F)], (C) *Ackr4* deficiency in the skin only (skin ACKR4 KO) [dashed lines in (E) and F)], and (D) combined *Ackr4* deficiency in skin and SLN (global ACKR4 KO) [dashed lines in (E) and F)]. The inclusion of 5 nM CCL19 and CCL21 in afferent lymph was used to simulate the levels of cytokines associated with *Ackr4* deficiency in the skin. Plots in rows (E) (IFR) and (F) (BF–TC border) are concentration profiles along the arrows indicated in Fig. 1D. Note in (E) and (F) the WT control and LN ACKR4 KO lines are indistinguishable (solid lines), whereas skin ACKR4 KO and global ACKR4 KO are overlapping (dashed lines).

IFR CCL21 gradients are disrupted in the SLNs of *Ackr4*-deficient mice due to the accumulation of excess extracellular CCL21 (7), yet our PVA indicated that variation in ACKR4 activity on the SCS ceiling has no impact on gradient formation in LNs. Indeed, intranodal distributions of CCL19, CCL21u, and CCL21b were unaffected by the complete removal of ACKR4 from baseline simulations (Fig. 6A, 6B, 6E, 6F). However, this scenario, referred to in this study as “LN ACKR4 knockout (KO),” does not consider the impact that loss of ACKR4-mediated chemokine scavenging from the skin, a rich source of ACKR4 (17, 26), has on intranodal chemokine distribution; the excess CCL21 seen in the IFR of *Ackr4*-deficient SLN might originate in the skin and enter the SLN via afferent lymph. To explore this, we considered two scenarios: 1) “skin ACKR4 KO,” which resembled the baseline simulation (referred to in Fig. 6 as wild-type [WT]) except that CCL21 and CCL19 were added to afferent lymph to mimic loss of ACKR4 from the skin (i.e., *C*_in,CCL19_ = *C*_in,CCL21_ = 5 nM) (Fig. 6C); and 2) “global ACKR4 KO,” where simulations were similar to 1) but in which ACKR4 was also absent from the SCS ceiling (Fig. 6D), mimicking global loss of ACKR4. These two simulations produced identical CCL19, CCL21u, and CCL21b contour maps in which most of the intranodal chemokine gradients seen in WT and LN ACKR4 KO were substantially altered (Fig. 6). CCL21b concentrations were higher in the IFR and the CCL21b gradient was virtually eliminated, akin to the IFR distribution of CCL21 seen experimentally in *Ackr4*-deficient SLN (7). In addition, the CCL19 IFR gradient directly under the SCS was reversed. The CCL21b and CCL19 gradients at the BF–TC border were less markedly affected, but a new CCL19 gradient was present in the BF under the SCS. At both of these locations the directionality and magnitude of the CCL21u gradient was unchanged, although there was an increase in CCL21u concentration throughout the LN. Further simulations incorporating lower concentrations of chemokine in afferent lymph resulted in similarly shaped chemokine profiles, with magnitudes varying monotonically between those seen for the global ACKR4 KO and WT (data not shown).

### Disrupting lymph flow alters intranodal chemokine distribution and unmasks a role for ACKR4 on the SCS ceiling

**FIGURE 7.**
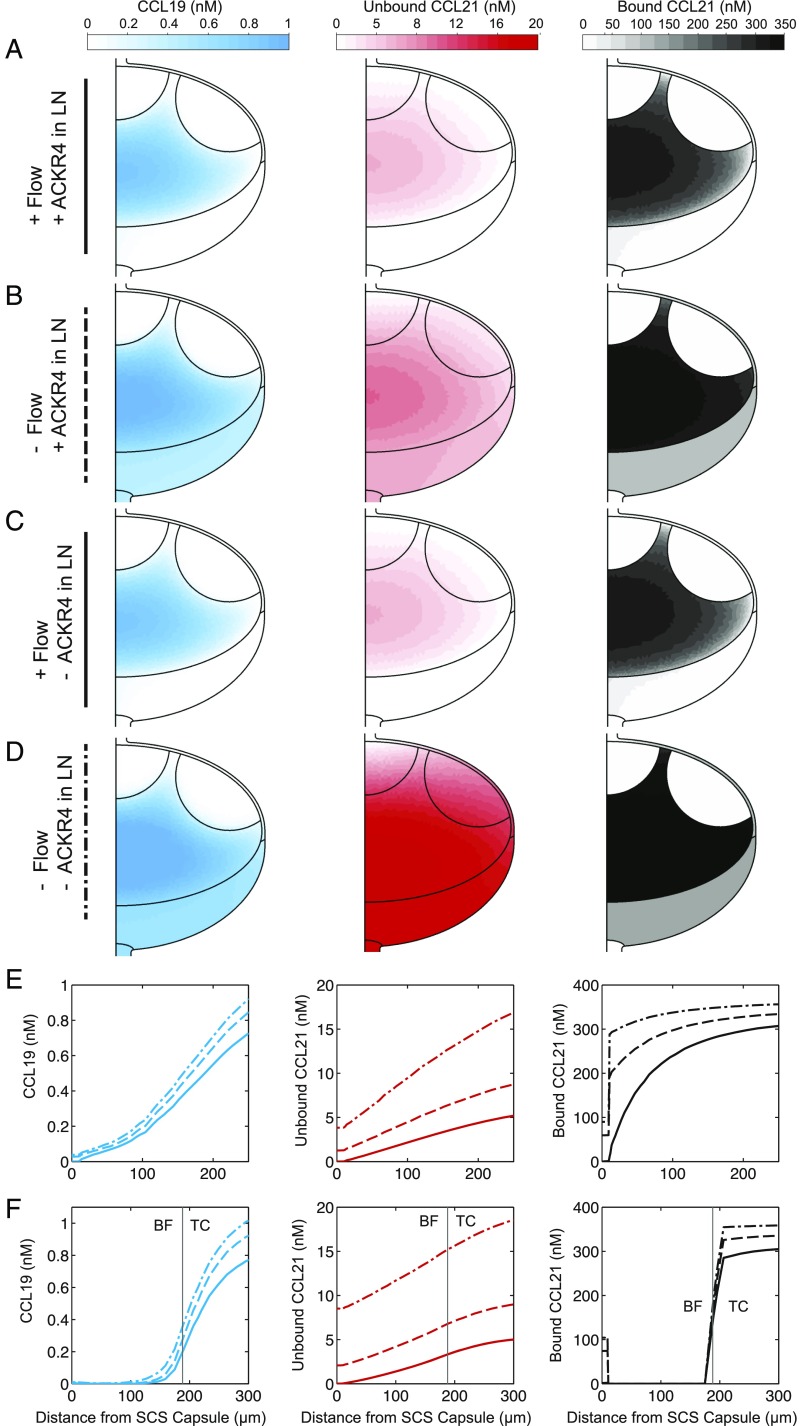
Disrupting lymph flow alters intranodal chemokine distributions and unmasks a role for ACKR4 on the SCS. Contours (**A**–**D**) and plotted gradients (**E** and **F**) of CCL19 (left column, blue), CCL21u (middle column, red), and CCL21b (right column, black) in the following simulations: (A) baseline flow in the presence of ACKR4 [solid lines in (E) and (F)], (B) 1% baseline flow in the presence of ACKR4 [dashed lines in (E) and (F)], (C) baseline flow in the absence of ACKR4 [solid lines in (E) and (F)], and (D) 1% baseline flow in the absence of ACKR4 [dash–dot lines in (E) and (F)]. Plots (E) and (F) are concentration profiles along the arrow indicated in Fig. 1D. Note the +Flow+ACKR4 and +Flow−ACKR4 lines are indistinguishable at these plot scales.

Next we explored the role of lymph flow in shaping intranodal gradients by performing simulations in which the flow rate was decreased to 1% of the baseline (Fig. 7A, 7B, 7E, 7F). This resulted in an increase in the concentration of all the chemokines throughout the TC, and although there were minimal changes in the magnitude and directionality of the CCL19 and CCL21u gradients, the CCL21b gradient in the IFR was noticeably reduced. There was also a marked accumulation of CCL21u, and particularly CCL21b, in the SCS; the concentration of all three chemokines was increased in the medulla; and CCL19 and CCL21u were present in efferent lymph. Thus, the model predicts that lymph flow shapes intranodal chemokine gradients and contributes to the clearance of chemokines from the SCS, medulla, and efferent lymph.

We wondered whether ACKR4 might limit the accumulation of chemokines in the SCS seen at low flow rates. Thus, we performed a further simulation in which we removed ACKR4-mediated scavenging from the SCS while simultaneously decreasing the flow rate to 1% of the baseline (Fig. 7D–F). Interestingly, this exaggerated the changes in chemokine distribution seen with low flow rate alone (Fig. 7B, 7E, 7F), contrasting the lack of effect seen when ACKR4 was removed in the context of baseline flow rates (Fig. 7C, 7E, 7F). Most notable were the increased accumulation of chemokines in the medulla and efferent lymph; the further reduction in magnitude of the CCL21b gradient in the IFR; and the marked increase in CCL21u concentration in the TC, BF, and SCS. These data indicate that ACKR4-mediated scavenging at the SCS ceiling is dispensable at times of efficient lymph flow, but helps stabilize intranodal chemokine distribution when lymph flow is compromised.

## Discussion

The mechanisms that control extracellular chemokine distribution are of significant immunological importance but they are currently poorly understood. Computational modeling offers a valuable enhancement to experimental approaches, and can be exploited to predict the probable outcomes of technically challenging or prohibitively expensive scenarios. Moreover, whereas reductionist experimental approaches are well suited for investigating individual mechanisms, modeling can integrate all relevant mechanisms to build a deeper understanding of how the whole system functions. To our knowledge, we have developed the first mathematical model of LN chemokine transport to include the important effects of lymph flow and fluid exchange with blood vessels, as well as incorporating binding dynamics between chemokines, receptors, and ECM. The proper construction and initial validation of such a model is firmly grounded in experimental evidence, but importantly the model we have developed has allowed us to predict the key processes regulating chemokine distribution and gradient formation in LNs as well as generate new testable hypotheses and provide alternative interpretations of existing experimental data.

Two key factors build confidence in the reliability of our model to predict the location of intranodal chemokine gradients. First, despite substantial changes to the input data in the PVA, the gradients predicted in the baseline simulation were robust in that they were always present in the IFR, BF, and BF–TC border, and their directionality was unchanged. Thus, any inaccuracies in the input parameters used in the baseline simulation might result in imprecise predictions of gradient magnitude, but they will not impact on the model’s ability to predict their location or directionality. Second, CCL21 gradients similar to those predicted have been detected in the IFR and BF by immunostaining WT LN sections with anti-CCL21 Abs (6, 7). This approach presumably detects ECM-bound chemokine (CCL21b), because most, if not all, unbound chemokine will be lost during tissue preparation and processing. Moreover, it remains technically very challenging to detect extracellular chemokine gradients in situ, and to our knowledge this has only been achieved for CCL21 in mice, presumably because of its very strong affinity for the ECM. This emphasizes the need for alternative approaches, including computational modeling.

The CCR7-dependent egress of DCs from the SCS (11) likely requires CCL21 bound to LECs on the SCS floor; any unbound chemokine in the SCS will be washed away by the dominant fluid flow toward the medulla. Immediately after DC egress, CCL21b in the IFR would be expected to activate integrin-mediated adhesion of DCs to fibroblastic reticular cells, and perhaps give some early directionality to the cells. However, our data show that the very high CCL21b concentrations in the IFR and TC mean that virtually all CCR7 receptors are occupied by CCL21b. This high occupancy, which in an LN would only occur on parts of the DC in direct contact with CCL21b-coated surfaces, would be expected to prevent DCs from sensing CCL21b concentration differences across its length. However, elegant in vitro experiments have shown that gradients of unbound CCR7 ligands can steer DCs adhered to surfaces by CCL21b-mediated integrin activation (15). DCs can therefore interpret information transmitted by unbound ligands through CCR7 even when CCL21b occupies all the CCR7 molecules on the adhered surface. Thus, in the IFR, CCL21b-rich surfaces might be responsible for DC adherence, but the gradients of CCL19 and/or CCL21u observed in the model, rather than those of CCL21b, could be responsible for steering DCs toward the TC.

The absence of CCL21b from the BF means that, in contrast to cells in the TC and IFR, most CCR7 molecules on cells in this area are unoccupied. Naive B cell homing to BFs requires CXCR5, which is activated by CXCL13 on follicular stromal cells (48). However, B cell migration within BFs depends on integrated responses through CXCR5, CCR7, and EBI2, a receptor for oxysterols (thought to form gradients in the BF toward the SCS) (49, 50), and it will be of interest to incorporate CXCL13, oxysterols, and their receptors into the computational model. Experimental approaches have revealed that CCR7 draws naive and Ag-activated B cells toward the BF–TC border, EBI2 attracts them toward the SCS, and CXCR5 aids their retention in the BF. Dynamic changes in the balance of activity of EBI2 and CCR7 enable naive B cells to patrol the BF and directs Ag-activated B cells first toward the SCS (to enhance Ag capture) and then to the BF–TC boundary (to search for cognate follicular helper T cells). Thus, *Ccr7*-deficient naive B cells accumulate near the SCS-proximal region of the BF, whereas artificially increasing CCR7 expression causes B cells to enter the TC, and CXCR5 overexpression prevents CCR7-driven, Ag-induced B cell movement to the BF–TC border (10).

The integration of our modeling outputs with these experimental findings leads to new hypotheses concerning the role of CCR7 ligands in the BF. The first is that a gradient of CCL21u is a major factor controlling CCR7-mediated B cell movement in the BF because it reaches deep into the BF from the TC. Testing this hypothesis is challenging given the profound impact of *Ccl21* deficiency on LN structure and function (51), and our inability to distinguish the functions of CCL21u from those of bound CCL21 in vivo. The second hypothesis is that CCL19-induced desensitization and internalization of CCR7 regulates cell behavior at the BF–TC border. This is more amenable to experimental testing because *Ccl19* deficiency has no detectable impact on LN structure (18, 23). For naive B cells, which have relatively low levels of CCR7, we hypothesize that a CCL19-induced reduction in CCR7 activity at the BF–TC border will allow signals through CXCR5 and EBI2 to dominate and that this will direct cells away from the BF–TC border to scan for Ag elsewhere in the BF. For Ag-activated B cells, which have higher levels of CCR7, a brake on CCR7 activity near the BF–TC border could hypothetically prevent CXCR5-mediated BF retention signals from being over-ridden by CCL21b-mediated signals through CCR7, thereby helping retain these cells at the BF–TC border. These hypotheses are testable using two-photon microscopy and immunohistology to track the movement and localization, respectively, of adoptively transferred Ag-specific B cells in the PLNs of WT and *Ccl19*-deficient recipients before and after immunization. Ab responses should be carefully examined in *Ccl19*-deficient mice, particularly after immunization with low doses of Ag when the precise localization of naive and activated B cells might be expected to be of most importance.

Other significant outputs from the model relate to the role of lymph flow and ACKR4. During normal lymph flow, ACKR4-mediated scavenging by LECs on the SCS ceiling had no effect on gradients, and PVA found no correlation between LN ACKR4 levels and the magnitude of the intranodal gradient. However, simulating loss of ACKR4-mediated scavenging from upstream tissue generated a distribution of CCL21 resembling that seen in *Ackr4*-deficient mice (7). The gradients of unbound chemokines in the IFR and BF were also substantially altered in these simulations. CCL19 and CCL21 are present in skin-draining afferent lymph of humans at concentrations of 0.016 and 0.011 nM, respectively (37), but these chemokines have not been measured in afferent lymph in WT or *Ackr4*-deficient mice. However, ACKR4 has been shown to limit the transport of endogenous CCL21 from peripheral tissues to SLNs in vivo by experiments demonstrating that SLNs from *Ackr4*-deficient *plt*/*plt* mice contain more CCL21 than SLNs from *plt*/*plt* mice (7). The *plt* mutation deletes *Ccl19* and the *Ccl21* gene responsible for producing CCL21 in LNs, but the *Ccl21* gene that generates CCL21 in nonlymphoid tissues remains intact (51–53). Thus, the excess CCL21 in the SLN of *Ackr4*-deficient *plt*/*plt* mice must be of nonlymphoid origin.

Lymph flow rate emerged as a key regulator of chemokine distribution in the model. Thus, in the baseline simulation, any unbound chemokine leaving the LN parenchyma is washed away in lymph rather than being scavenged by ACKR4. However, low lymph flow simulations unmasked a potential role for ACKR4 on the SCS ceiling: under these conditions ACKR4 clearly stabilized intranodal gradients and limited chemokine accumulation in the LN and efferent lymph. Physiologically, ACKR4 on the SCS ceiling might be important in stabilizing chemokine distribution during the early inflammatory phases of immune responses when lymph flow from the affected upstream tissues is diminished and these tissues swell.

These observations present several additional new hypotheses. The first is that, under conditions of normal lymph flow, ACKR4 in the skin, rather than on the ceiling of the SCS, regulates chemokine gradients and DC trafficking in SLNs. This could be tested using mice in which *Ackr4* alleles can be conditionally deleted. Although currently unavailable, they would allow *Ackr4* to be specifically removed from keratinocytes, the dominant source of ACKR4 in the skin; or from LECs, the only cell type expressing ACKR4 in LNs. Alternatively, LN transplantation experiments could be undertaken between WT and *Ackr4*-deficient mice. Interestingly, in contrast to the SLN (7, 17), CCR7-dependent DC trafficking to the gut-draining mesenteric LN is unaffected by *Ackr4* deficiency, either at steady state or in response to inflammation-induced mobilization (R.J.B. Nibbs, unpublished observations). This is despite skin-draining and mesenteric LNs both making large quantities of CCL21 and both expressing ACKR4 on LECs lining the SCS ceiling (R.J.B. Nibbs, unpublished observations). These observations are consistent with the idea that it is the characteristics of the tissue upstream that are critical in determining the impact of *Ackr4* deficiency on chemokine gradients and leukocyte trafficking in draining LNs.

The second hypothesis is that intranodal gradients of CCL21u and CCL19 can, like those of bound CCL21, be disrupted in *Ackr4*-deficient SLNs by excess chemokine entering into the lymph. It is not possible to visualize these gradients or distinguish the functions of CCL21u from those of bound CCL21 in vivo, but, by generating *Ackr4*/*Ccl19* doubly deficient mice, it is possible to determine if CCL19 contributes to aberrant migration into or within LNs caused by *Ackr4* deletion. Indeed, *Ccl19* deletion rescues the defective departure of DCs from the inflamed skin of *Ackr4*-deficient mice (17).

The third hypothesis is that reducing lymph flow rate will alter CCL19/CCL21 distribution and the migratory behavior of CCR7^+^ leukocytes in LNs, and that this will be particularly evident in *Ackr4*-deficient mice. To test this, lymph flow into LNs of WT and *Ackr4*-deficient mice would need to be stopped or reduced; this is technically challenging but could theoretically be achieved by the ligation or partial ligation of some or all of the afferent lymphatic vessels entering a specific LN. IFR gradients of bound CCL21 could then be visualized and quantified (7); the levels of CCL19 and CCL21 in efferent lymph could be measured; and the migratory behavior of adoptively transferred, fluorescently labeled leukocytes at specific intranodal locations could be assessed by two-photon microscopy. These experiments would need to be carefully controlled given that lymph and lymph-borne DCs are known to control lymphocyte trafficking by regulating the structure and function of high endothelial venules (54–57), and that migratory DCs can cleave CCL21b to release a soluble form (15). Moreover, lymph flow–induced wall shear stresses in the range of those present in collecting lymphatics regulate LEC signaling by modulating NO production (58, 59), histamine release (60), and calcium dynamics (61), and further studies are required to determine if shear stress alters chemokine production and ACKR4 function in LECs. Wall shear stresses in the SCS are in the same range as those in collecting lymphatics (28). However, these effects may depend on nodal LEC location because, in our model of lymph flow in PLN (28), shear stress under basal afferent flow ranges from >5 dyn/cm^2^ near the Af to <1 dyn/cm^2^ closer to the medulla.

The model predictions need to be considered in the context of its limitations. We made some necessary simplifications that did not take into account the inherent heterogeneity in LNs. We assumed, for example, that chemokine production, cell population density, and receptor availability was uniform across each LN region. The effects of leukocyte recruitment, migration, and departure were also not included; although the model could be coupled to models of cell migration to investigate cellular chemotaxis and haptotaxis in the LN (62, 63). Nonetheless, to our knowledge, the inclusion of the number of physical and biological phenomena shown in Fig. 1 makes this the most thorough model characterization in the literature. Previous models captured only subsets of these phenomena as parts of efforts to characterize simplified reductionist experiments (4, 5, 28, 64, 65), or actions of single cells in simplified environments (29, 31, 66, 67).

The reliability of the model outputs also clearly depends on the accuracy of the input data. Many input parameters were collected from experimental data, but these may not precisely reflect circumstances in vivo. Other parameters used have not been measured experimentally, or are currently impossible to measure, so sensible estimates had to be included. The PVA helps determine which parameters are likely to be the key regulators of the model outputs and their accurate measurement, using primary sources of tissue or cells, is a priority for future refinement of the model. As discussed above, gradient location and directionality was robust in the PVA, but, not unexpectedly, variation in ECM binding site availability, effective diffusivity, and production of CCL19 or CCL21 emerged as the key parameters that strongly correlated with changes in gradient magnitude, total amount of chemokine in the LN, and/or chemokine concentration in efferent lymph. Interestingly, there was a strong negative correlation between the number of ACKR4 receptors on the SCS ceiling and the concentration of CCL19 and CCL21 in efferent lymph. This was not apparent when we removed ACKR4 from the baseline simulation, presumably because the input parameters we used in this simulation did not result in enough chemokine entering the SCS or efferent lymph for an effect of ACKR4 loss to be observed. Nonetheless, it emphasizes that a role for intranodal ACKR4 under normal lymph flow conditions cannot be excluded, at least in terms of regulating chemokine in efferent lymph, and it will be of interest to see if CCL19 and CCL21 are elevated in the efferent lymph of unchallenged *Ackr4*-deficient mice. If so, this could affect the behavior of lymph-borne leukocytes and/or modify chemokine gradients in LNs further down the LN chain in a manner akin to that discussed above for chemokine entering LNs from nonlymphoid tissue.

In summary, by integrating our knowledge of lymph transport in LNs with multiple biochemical processes describing the CCL19–CCL21–CCR7–ACKR4 network, we have built a comprehensive model of CCL19 and CCL21 transport and gradient formation in the LN. The model reliably reproduces the available experimental data, but more importantly has offered alternative interpretations of experimental data and created new testable hypotheses. It has identified the key physical and biochemical parameters that are likely to be critically important in controlling chemokine distribution and gradient formation, and, by extension, leukocyte migration and immune responses. Incorporating additional, and more accurate, experimental data will bring further power to the model. These data will come from the many experiments discussed above, but it will also be important to analyze primary LN cells to more precisely quantify key model parameters, such as the number of surface CCR7 molecules on LN leukocytes, the rate of CCL19/21 production by stromal cells, and the efficiency of ACKR4-mediated scavenging by SCS ceiling LECs. Including other chemokines and their receptors, such as CXCL13/CXCR5 and oysterols/EBI2, will also be of interest and will allow competing intranodal gradients to be considered. Moreover, our computational model could be readily employed to investigate gradient formation in other tissues and as a tool to identify key factors regulating leukocyte migration in health and disease.

## Supplementary Material

Data Supplement
