# Supplementary material for: A Novel Computational Model Predicts Key Regulators of Chemokine Gradient Formation in Lymph Nodes and Site-Specific Roles for CCL19 and ACKR4
Source: J Immunol. 2017 Aug 14;199(7):2291–304. doi: 10.4049/jimmunol.1700377 (PMC5602158; doi:10.4049/jimmunol.1700377)
Supplement: Data Supplement [file JI_1700377.zip › JI_1700377_Supplemental_Figures_1.pdf]

A Novel Computational Model Predicts Key Regulators of Chemokine Gradient Formation in Lymph Nodes and Site-Specific Roles for CCL19 and ACKR4.

M. Jafarnejad, B.S. Brook, D.C. Zawieja, R.J.B. Nibbs, J.E. Moore Jr.

Supplementary Information

Governing equations

$$\frac{\partial}{\partial t}U_{21} = \underbrace{D_{21}\nabla^2 U_{21}}_{\text{diffusion}} - \underbrace{\mathbf{u}\cdot\nabla U_{21}}_{\text{advection}} - \underbrace{J_{BV,21}}_{\text{flux into BV}} + \underbrace{\Pi_{21}}_{\text{production}} - \underbrace{\lambda_{\text{on}} U_{21} R_7 + \lambda_{\text{off}} U_{21\text{-}R7}}_{\text{binding to/unbinding from CCR7}}$$
$$- \underbrace{k_1 (E - B_{21}) U_{21} + k_2 B_{21}}_{\text{binding to/unbinding from ECM}} \quad (1)$$

$$\frac{\partial}{\partial t}U_{19} = D_{19}\nabla^2 U_{19} - \mathbf{u}\cdot\nabla U_{19} - J_{BV,19} + \Pi_{19} - \lambda_{\text{on}} U_{19} R_7 + |\lambda_{\text{off}} U_{19\text{-}R7} \quad (2)$$

$$\frac{\partial}{\partial t}B_{21} = -\lambda_{\text{on}} B_{21} R_7 + \lambda_{\text{off}} B_{21\text{-}R7} + k_1 (E - B_{21}) U_{21} - k_2 B_{21} \quad (3)$$

$$\frac{\partial R_7}{\partial t} = -\lambda_{\text{on}} R_7 (U_{21} + B_{21} + U_{19}) + \lambda_{\text{off}} (U_{21\text{-}R7} + B_{21\text{-}R7} + U_{19\text{-}R7}) + \lambda_{\text{up}} U_{19\text{-}R7\text{int}} \quad (4)$$

$$\frac{\partial}{\partial t}U_{19\text{-}R7} = \lambda_{\text{on}} R_7 U_{19} - \lambda_{\text{off}} U_{19\text{-}R7} - \lambda_{\text{des}} U_{19\text{-}R7} \quad (5)$$

$$\frac{\partial}{\partial t}U_{19\text{-}R7\text{des}} = \lambda_{\text{des}} U_{19\text{-}R7} - \lambda_{\text{int}} U_{19\text{-}R7\text{des}} \quad (6)$$

$$\frac{\partial}{\partial t}U_{21\text{-}R7} = \lambda_{\text{on}} R_7 U_{21} - \lambda_{\text{off}} U_{21\text{-}R7} \quad (7)$$

$$\frac{\partial}{\partial t}B_{21\text{-}R7} = \lambda_{\text{on}} R_7 B_{21} - \lambda_{\text{off}} B_{21\text{-}R7} \quad (8)$$

$$R_{7,\text{tot}} = R_7 + U_{21\text{-}R7} + B_{21\text{-}R7} + U_{19\text{-}R7} + U_{19\text{-}R7\text{des}} + U_{19\text{-}R7\text{int}} \quad (9)$$

$$J_{BV,i} = J_v(1-\sigma_f)\left(\frac{U_i + U_{BV,i}}{2}\right) + P_u S(U_i - U_{BV,i}), \qquad i = 21, 19 \quad (10)$$

Boundary conditions at capsule boundary

$$\frac{\partial}{\partial t}U_{19\text{-}A4} = \eta_{\text{on}} U_{19} A_4 - \eta_{\text{off}} U_{19\text{-}A4} - \eta_{\text{int}} U_{19\text{-}A4} \quad (11)$$

$$\frac{\partial}{\partial t}U_{19\text{-}A4\text{int}} = \eta_{\text{int}} U_{19\text{-}A4} - \eta_{\text{up}} U_{19\text{-}A4\text{int}} \quad (12)$$

$$\frac{\partial}{\partial t}U_{21\text{-}A4} = \eta_{\text{on}} U_{21} A_4 - \eta_{\text{off}} U_{21\text{-}A4} - \eta_{\text{int}} U_{21\text{-}A4} \quad (13)$$

$$\frac{\partial}{\partial t}U_{21\text{-}A4\text{int}} = \eta_{\text{int}} U_{21\text{-}A4} - \eta_{\text{up}} U_{21\text{-}A4\text{int}} \quad (14)$$

$$A_{4,\text{tot}} = A_4 + U_{21\text{-}A4} + U_{21\text{-}A4\text{int}} + U_{19\text{-}A4} + U_{19\text{-}A4\text{int}} \quad (15)$$

$$\mathbf{n}\cdot\nabla U_i = \eta_{\text{int}} U_{i\text{-}A4}, \qquad i = 19, 21 \quad (16)$$

Parameter estimation

$$X_i = \sum_k N_k P_{k,j} EV_{X,k} \frac{X^{\max}}{N_A} \frac{Vol_i}{Vol_j} \quad (17)$$

Chemokine, receptor or complex	Abbreviations used in the text	Abbreviations of the concentration used in the equations
Unbound CCL21	CCL21u	U_{21}
ECM bound CCL21	CCL21b	B_{21}
Unbound CCL19	CCL19	U_{19}
Unbound CCR7	CCR7	R_7
Unbound ACKR4	ACKR4	A_4
Unbound CCL21 bound to CCR7	CCL21u-CCR7	$U_{21\text{-}R7}$
ECM bound CCL21 bound to CCR7	CCL21b-CCR7	$B_{21\text{-}R7}$
Unbound CCL19 bound to CCR7	CCL19-CCR7	$U_{19\text{-}R7}$
Internalized CCL19-CCR7	CCL19-CCR7_{int}	$U_{19\text{-}R7\text{int}}$
Desensitized CCL19-CCR7	CCL19-CCR7_{des}	$U_{19\text{-}R7\text{des}}$
Unbound CCL21 bound to ACKR4	CCL21u-ACKR4	$U_{21\text{-}A4}$
Internalized CCL21u-ACKR4	CCL21u-$\text{ACKR4}_{\text{int}}$	$U_{21\text{-}A4\text{int}}$
Unbound CCL19 bound to ACKR4	CCL19-ACKR4	$U_{19\text{-}A4}$
Internalized CCL19-ACKR4	CCL19-$\text{ACKR4}_{\text{int}}$	$U_{19\text{-}A4\text{int}}$

Figure S1. Equations used in modelling and chemokine/receptor abbreviations
The equation number referred to in the article text is shown to the right of each equation. The table summarises the abbreviations used in the equations to represent chemokine, receptor and chemokine/receptor complexes. Parameter abbreviations and definitions are shown in Figure S2.

Parameter	Definition	Value	Reference
k_1	On-rate constant for ECM binding	0.000093 $nM^{-1}s^{-1}$	(31)
k_2	Off-rate constant for ECM binding	0.00012 s^{-1}	(31)
$N_{E,\max}$	Maximum ECM binding sites per cell	1,000,000 #/cell	(31)
λ_{on}	On-rate constant for CCR7 binding	0.001 $nM^{-1}s^{-1}$	(29, 30)
λ_{off}	Off-rate constant for CCR7 binding	0.005 s^{-1}	(29, 30)
λ_{des}	Desensitization rate constant for CCR7 complexes	0.003 s^{-1}	(29, 30)
λ_{int}	Internalization rate constant for CCR7 complexes	0.0005 s^{-1}	(29, 30)
λ_{up}	Resurfacing rate constant of CCR7	0.000375 s^{-1}	(29, 30)
$N_{R7,\max}$	Maximum CCR7 per cell	30,000 #/cell	Estimated
η_{on}	On-rate constant for ACKR4 binding	1.0 s^{-1}	(27)
η_{off}	Off-rate constant for ACKR4 binding	0.002 s^{-1}	(27)
η_{in}	Internalization rate constant for ACKR4 complexes	0.5 $nM^{-1}s^{-1}$	(27)
η_{up}	Resurfacing rate constant for ACKR4	2.25 s^{-1}	(27)
$N_{A4,\max}$	Maximum ACKR4 per cell	30,000 #/cell	Estimated
P_u	Average permeability of blood vessels to chemokines	$5x10^{-7}$ cm/s	(41, 42)
$U_{\mathrm{BV,19}}$	Average unbound CCL19 concentration in blood vessels	0.01 nM	(36, 37)
$U_{\mathrm{BV,21}}$	Average unbound CCL21 concentration in blood vessels	0.01 nM	(36, 37)
σ_{f}	Filtration reflection coefficient	0	(41)
S	Average surface area density	33000 m^{-1}	(28)

Figure S2. Parameter abbreviations and definitions

A

Cell types	CCL19		CCL21		CCR7		Matrix Binding site				
							Transmembrane ‡		Extracellular §		Average
	Exp value*	Norm *EV* †	Exp value	Norm *EV*	Exp value	Norm *EV*	Exp value	Norm *EV*	Exp value	Norm *EV*	
B cell	0	0	0	0	1000	0.25	0	0	0	0	0
T cell	0	0	0	0	2000	0.50	0	0	0	0	0
DC	0	0	0	0	4000	1.00	1500	0.35	0	0	0.17
MΦ	0	0	0	0	0	0	0	0	0	0	0
BEC	150	0.02	9200	0.66	0	0	1000	0.23	7000	0.74	0.48
LEC	300	0.03	1500	0.11	0	0	1200	0.27	5400	0.57	0.42
FRC	8800	1.00	14000	1.00	0	0	4300	1.00	9400	1.00	1.00

B

Cell type	percentage of the cells in each region ($P_{k,j}$)				Total cells (N_k)	Reference
	B cell follicle	T cell paracortex	SCS	Medulla		
B cell	90	0	0	10	933,000	(7, 43, 44)
T cell	0	90	0	10	1,000,000	(7, 43, 44)
DC	0	100	0	0	68,500	(7, 43, 44)
MΦ	0	0	10	90	120,000	(7)
BEC	0	75	0	25	14,000	(40, 43, 45)
LEC	0	0	10	90	15,000	(45)
FRC	0	100	0	0	28,500	(43, 45)

C

Parameter	Unit	Baseline value	Lower bound	Upper bound
Maximum matrix binding sites	#/cell	1,000,000	200,000	1,800,000
Maximum CCL19 production	#/cell-s	4.11	0.8	7.4
Maximum CCL21 production	#/cell-s	6.54	1.3	11.8
Maximum CCR7 per cell	#/cell	30000	6000	54000
Maximum ACKR4 per cell	#/cell	30000	6000	54000
Ratio of effective diffusivity to diffusivity in water	-	0.10	0.025	0.4

Figure S3. Parameter estimates.

A: *Normalized expression of key genes by different cell types in the LN.* Data were generated using the ImmGen database (35, 36). * Exp value: RNA expression values from ImmGen; † Norm *EV*: expression value normalized by the maximum; ‡ Data from syndecans 1-4 and glypicans 1-6 are summed to calculate the expression value; § Data from perlecan, agrin, biglycan and collagen IV are summed to calculate the expression value

B: *Estimates of total cell numbers (N_k) and the percentage of cell types ($P_{k,j}$) present in different regions of the LN.*

C: *Input parameter range for Latin hypercube sampling.*
